# Accelerated evolution associated with genome reduction in a free-living prokaryote

**DOI:** 10.1186/gb-2005-6-2-r14

**Published:** 2005-01-14

**Authors:** Alexis Dufresne, Laurence Garczarek, Frédéric Partensky

**Affiliations:** 1 Station Biologique, UMR 7127 CNRS et Université Paris 6, BP74, 29682 Roscoff Cedex, France

## Abstract

**Background:**

Three complete genomes of *Prochlorococcus *species, the smallest and most abundant photosynthetic organism in the ocean, have recently been published. Comparative genome analyses reveal that genome shrinkage has occurred within this genus, associated with a sharp reduction in G+C content. As all examples of genome reduction characterized so far have been restricted to endosymbionts or pathogens, with a host-dependent lifestyle, the observed genome reduction in *Prochlorococcus *is the first documented example of such a process in a free-living organism.

**Results:**

Our results clearly indicate that genome reduction has been accompanied by an increased rate of protein evolution in *P. marinus *SS120 that is even more pronounced in *P. marinus *MED4. This acceleration has affected every functional category of protein-coding genes. In contrast, the 16S rRNA gene seems to have evolved clock-like in this genus. We observed that MED4 and SS120 have lost several DNA-repair genes, the absence of which could be related to the mutational bias and the acceleration of amino-acid substitution.

**Conclusions:**

We have examined the evolutionary mechanisms involved in this process, which are different from those known from host-dependent organisms. Indeed, most substitutions that have occurred in *Prochlorococcus *have to be selectively neutral, as the large size of populations imposes low genetic drift and strong purifying selection. We assume that the major driving force behind genome reduction within the *Prochlorococcus *radiation has been a selective process favoring the adaptation of this organism to its environment. A scenario is proposed for genome evolution in this genus.

## Abstract 2

*Prochlorococcus* sp. are marine bacteria with very small genomes. The mechanisms by which these reduced genomes have evolved appears, however, to be distinct from those that have led to small genome size in intracellular bacteria.

## Background

The size of bacterial genomes is primarily the result of two counteracting processes: the acquisition of new genes by gene duplication or by horizontal gene transfer; and the deletion of non-essential genes. Genomic flux created by these gains and losses of genetic information can substantially alter gene content. This process drives divergence of bacterial species and eventually adaptation to new ecological niches [1]. In some cases, gene deletion may prevail over gene acquisition, leading to genome reduction. This process has occurred several times during evolution and has been well documented for cellular organelles [2,3], obligate pathogens such as *Mycoplasma genitalium *[4] or phytoplasmas [5] and symbionts such as the insect endosymbiont *Buchnera *[6-8] or the hyperthermophile *Nanoarchaeum equitans *[9]. In the case of organelles, the degree of genome reduction can be extensive as a result of massive gene transfer into the host nucleus, allowing maintenance of the corresponding functions in the resulting composite organism. Mitochondrial or chloroplast genomes, for instance, can be as small as 6 kilobases (kb) [10] and 35 kb [11], respectively. In the case of obligate host-dependent bacteria, the reduction is more limited because the relationships with their hosts are less intimate than for organelles in eukaryotic cells. Thus, obligatory pathogens need to retain a minimum of functions that allow them to infect new hosts and to avoid host defenses, and obligate endosymbionts carry genes which are absolutely necessary for host survival. For instance, a substantial part (approximately 10 %) of the *Buchnera *genome is devoted to biosynthesis of amino acids which are essential to its host [6].

So far, all characterized examples of genome reduction have been associated with a change from a free-living to a host-dependent lifestyle [12]. It is therefore intriguing that a similar phenomenon of genome reduction has occurred within the free-living marine cyanobacterial genus *Prochlorococcus *[13-15]. The latter is present at high abundance (often over 10^5 ^cells/ml) in all nutrient-poor areas of the world's oceans between 40°N and 40°S and is probably the most abundant photosynthetic organism on Earth [16,17]. It has been shown that two major ecotypes exist within this genus [18]. The first is adapted to grow at the base of the illuminated layer and displays a high divinyl-chlorophyll *b *to *a *ratio; the second inhabits the upper layer of the ocean and has a low divinyl-chlorophyll *b *to *a *ratio [19]. The genome of one high-light-adapted (HL) strain, *Prochlorococcus marinus *MED4 [14], and of two low-light-adapted (LL) strains, *P. marinus *SS120 [13] and *Prochlorococcus *species MIT9313 [14], have recently been sequenced and annotated.

Phylogenetic trees based on 16S rRNA sequences [18] or 16S-23S ribosomal internal transcribed spacer sequences [20] show that *Prochlorococcus *sp. MIT9313 branches at the base of the *Prochlorococcus *radiation, close to the *Synechococcus *group [21]. In contrast, the *Prochlorococcus *HL clade, encompassing the MED4 strain, appears to be the most recently evolved *Prochlorococcus *group, consistent with the fact that this clade is much less diversified than are the LL clades.

Despite the close relatedness of these strains, their genomes vary widely in terms of size, G+C content and the number of protein-coding genes (Table 1). While the general characteristics of the MIT9313 genome are very similar to those of the *Synechococcus *sp. WH8102 genome [22], MED4 has the smallest genome for a photosynthetic organism known to date and the SS120 genome is only 90 kb larger. Furthermore, this genome reduction is clearly accompanied by a drift in G+C content, a phenomenon that commonly occurs during the evolution of host-dependent genomes [23]. However, the evolutionary mechanisms involved in the genome reductive process are most probably different from those that have occurred in host-dependent organisms. Using comparative sequence analyses of the four genomes of marine picocyanobacteria published to date, we have attempted to better understand the causes and consequences of this phenomenon and to address the relationships between genome reduction and niche adaptation in marine picocyanobacteria.

## Results

### Synteny and genome stability

**Figure 1:**
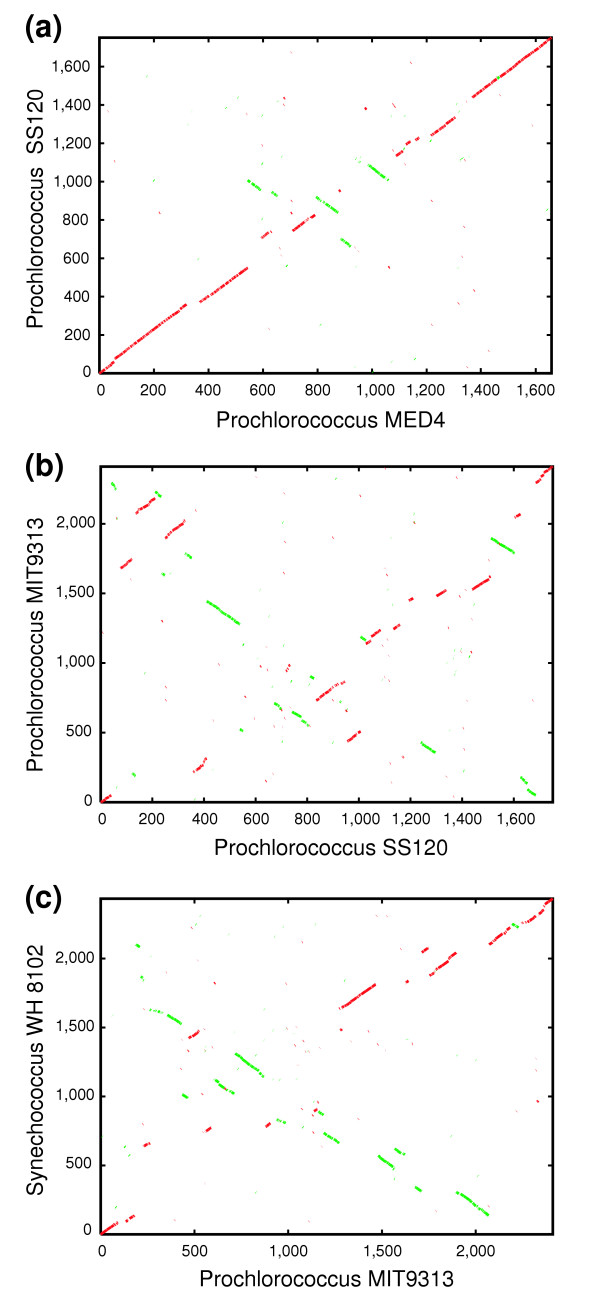
Alignments of complete genome sequences of marine picocyanobacteria. Genome sequences are translated in their six reading frames. **(a) **Comparison of the MED4 and SS120 genomes; **(b) **comparison of the SS120 and MIT9313 genomes; **(c) **comparison of the MIT9313 and WH8102 genomes. Colinear segments are shown in red and inversions in green. Translocated segments are above or below the diagonal.

Alignments of whole genomes show a strong conservation of the gene order between MED4 and SS120 (Figure 1a). There are only five inversions larger than 20 kb between these two genomes. In contrast, the large number of inversions and translocations and the shorter size of the colinear segments between SS120 and MIT9313 on the one hand and MIT9313 and WH8102 on the other hand (Figure 1b,c) indicate that extensive genome rearrangements have occurred not only between *Synechococcus *and *Prochlorococcus *but also between MIT9313 and the two other *Prochlorococcus *strains (see also Figure 2 in [14]). The degree of synteny observed between the four marine picocyanobacteria genomes strengthens the hypothesis of a more recent divergence of the clades containing MED4 and SS120 than of the clade containing MIT9313.

### Overall genome composition

The downsizing of MED4 and SS120 genomes during evolution is associated with a genome-wide adenine (A) and thymine (T) enrichment (Table 1). The bias is most pronounced at neutral sites such as intergenic regions (MED4, 76.6% A+T; SS120, 69.3% A+T) and third-codon positions of protein-coding genes (MED4, 79.7% A+T; SS120, 73.85% A+T). This bias has little effect on ribosomal RNA genes (5S, 16S and 23S) which have a G+C content greater than 50% in all four picocyanobacterial genomes. In both MED4 and SS120, the single rRNA gene cluster can easily be spotted as a G+C-rich anomaly compared to the rest of the genome (see for example, Figure 1 in [15]). In direct contrast, protein-coding genes are strongly affected by the extreme base composition of these genomes. First, the bias influences codon usage since, for a given amino acid, AT-rich codons are preferentially used (Figure 3a). Second, the amino-acid composition of the proteins themselves is affected (Figure 3b). Indeed, when compared to *Prochlorococcus *sp. MIT9313 and *Synechococcus *sp. WH8102, the genes of *P. marinus *MED4 and SS120 contain fewer amino acids encoded by G+C-rich codons (for example, alanine or arginine) and more amino acids encoded by A+T-rich codons (for example, isoleucine or lysine).

### Orthologous gene pool size

A total of 1,306 orthologs belonging to all major functional categories are common to the four genomes (see Additional data file 1) and probably constitute an estimate of the core of genes conserved in all marine picocyanobacteria. This is sensibly more than the pool of around 1,000 orthologs identified by W.R. Hess [15]. The difference certainly results from the use by the latter author of a low E-value threshold (10e^-12^) for BLAST comparisons. In contrast, our analysis is based on identification of reciprocal best hits without the use of any particular threshold (apart from the default BLAST threshold) and consequently allows the detection of orthologous relationships whatever the gene lengths or the level of similarity. Still, our ortholog identification process is rather strict and the set of orthologs identified in this study probably corresponds to a lower estimate of the actual number of orthologs shared by the four genomes. This set of genes represents a substantial percentage of the total pool of all protein-coding genes in *P. marinus *MED4 (73.2%) and SS120 (69.2%) and about half of the gene set in *Prochlorococcus *sp. MIT9313 (56.2%) and *Synechococcus *sp. WH8102 (51.1%). These percentages are consistent with the differences in the respective number of genes within these genomes (Table 1) and are compatible with the assumption that a massive gene loss has occurred in MED4 and SS120 during their evolution from a *Prochlorococcus *ancestor with a larger genome [13-15].

### Accelerated rate of evolution of protein-coding genes in *Prochlorococcus*

**Figure 2:**
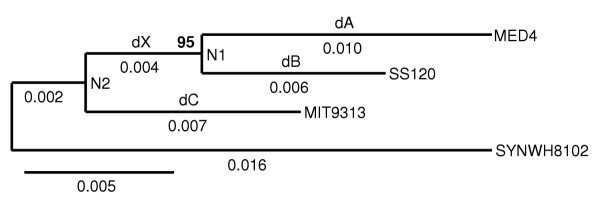
Phylogenetic tree of 16S rRNA genes from the four marine picocyanobacteria. Neighbor-joining tree with Kimura 2-parameter correction. The bootstrap value (1,000 replications) is shown in boldface. Lengths of the branches dA, dB, dC and dX (see text) are given below the branches. N1, node 1, branchpoint between MED4 and SS120; N2, node 2, branchpoint between MIT9313 and Node 1.

Because biased base composition seems to constrain amino-acid usage in the *Prochlorococcus *genomes, we have investigated whether it also affects the rate of protein sequence evolution in these genomes. We used the 1,306 orthologs common to the four genomes to estimate the amino-acid substitution rate in each genome. Branch lengths calculated for a given tree topology (the same topology as for the 16S rRNA gene tree; see Figure 2) are 0.46, 0.22, 0.16 and 0.14 amino acid substitutions per site for branches dA, dB, dC and dX, respectively. Using *Synechococcus *sp. WH8102 as the outgroup, we tested the rate-constancy hypothesis and computed the ratios of branch lengths. Relative rate tests (two-cluster and branch length tests) indicate that protein sequences evolved at significantly different rates (*P *< 0.001) between MED4, SS120 and MIT9313. Therefore the hypothesis of a constant evolutionary rate between these strains can be rejected for protein-coding genes. The calculation of branch-length ratios reveals that the amino-acid substitution rate is 2.04-fold higher inMED4 than in SS120 (dA/dB) and 3.81-fold higher in MED4 than in MIT9313 ((dA+dX)/dC). This rate is also 2.31-fold higher for SS120 than for MIT9313 ((dB+dX)/dC). Computation of branch lengths for each functional category shows that the increased rate of amino-acid replacement in protein sequences concerns every category (Figure 4 and Table 2). These results imply that the rate of amino-acid substitution increased during evolution of the *Prochlorococcus *genus concomitantly with genome reduction and increase in A+T content.

### Synonymous and nonsynonymous substitutions

The ratio of the rate of nonsynonymous substitutions (*d*_N_) to the rate of synonymous substitutions (*d*_S_) is commonly used to measure the relative rate of purifying selection acting at the protein level. We determined *d*_S _and *d*_N _for each gene pair of every group of orthologs and their values were averaged for each genome. Surprisingly, we observed saturation at synonymous sites for all genome pairs (*d*_S _> 2) and the calculation of the *d*_N_/*d*_S _ratio was thus impossible. Still, the average *d*_N _was higher between MED4 and SS120 (0.36) than between SS120 and MIT9313 (0.32). The lowest *d*_N _was observed between MIT9313 and WH8102 (0.24), a finding which is consistent with the relative acceleration of amino-acid substitutions in MED4 and in SS120.

### DNA-repair systems

A shift in base composition may reflect the loss of DNA-repair genes and we therefore determined the presence or absence of genes involved in these mechanisms. As the mutational pressure is toward a high A+T content in both MED4 and SS120, we looked more closely at those genes whose absence could increase the frequency of G:C to A:T mutations. Among the genes putatively encoding DNA-repair enzymes identified in MIT9313 and WH8102, a few are missing in SS120 and/or MED4 (Table 3). Both MED4 and SS120 lack the *ada *gene, which encodes 6-O-methylguanine-DNA methyltransferase, which repairs alkylated forms of guanine and thymine in DNA. Such alkylations generate lesions that can lead to G:C to A:T transversions [24]. Interestingly, the MED4 genome is the only one among the four picocyanobacteria not to encode the A/G-specific DNA glycosylase MutY, as previously noted by Rocap and co-workers [14]. This enzyme acts with MutT (NTP pyrophosphohydrolase) and MutM (formamido-pyrimidine-DNA glycosylase) in the GO system to avoid misincorporation of oxidized guanine (8-oxoG) in DNA and to repair the base mismatches A:8-oxoG [25]. In *Escherichia coli*, knocking out both *mutM *and *mutY *translates into a 1,000-fold increase of G:C to A:T transversions in comparison to the wild-type strain [26]. In addition to MutT and MutY, MIT9313 and WH8102 encode a third enzyme of the NUDIX hydrolase family that is missing in MED4 and SS120. This hydrolase could act to prevent mutations. However because of the broad substrate specificity of this family, one cannot know with certainty the function of this protein. Likewise, two genes coding for enzymes of the RecF pathway have been lost either by both MED4 and SS120 (DNA helicase RecQ) or only by MED4 (exonuclease RecJ).

## Discussion

The process of genome reduction which has occurred within the *Prochlorococcus *radiation has to our knowledge never been observed so far in any other free-living prokaryote. Since *Prochlorococcus *sp. MIT9313 has a genome size very similar to that of *Synechococcus *sp. WH8102 (2.4 megabase-pair (Mbp)), as well as several other marine *Synechococcus *spp. (M. Ostrowski and D. Scanlan, personal communication), it is reasonable to assume that the common ancestor of all *Prochlorococcus *species also had a genome size around 2.4 Mbp. Under this hypothesis, the genome reduction which has occurred in MED4 would correspond to around 31%. By comparison, the extent of genome reduction in the insect endosymbiont *Buchnera*, as compared to a reconstructed ancestral genome, is around 77% [27]. The genome of *P. marinus *SS120 - and *a fortiori *the MED4 genome - is considered to be near minimal for a free-living oxyphototrophic organism [13]. It would seem that genome reduction in these organisms probably cannot proceed below a certain limit, corresponding to a gene pool containing all the essential genes of biosynthetic pathways and housekeeping functions (probably including most of the 1,306 four-way orthologous genes identified in this study) plus a number of other genes, including genus-specific as well as niche-specific genes. For instance, MED4 encodes a number of photolyase-related proteins, a few specific ABC transporters (for cyanate, for example; [14] and data not shown). These specific compounds might be critical for survival in the upper water layer, which receives high photon fluxes, UV light and is nutrient-depleted, but less so for life deeper in the water column.

If both *Prochlorococcus *lineages and host-dependent organisms have undergone genome reduction associated with accelerated substitution rates, these phenomena must have arisen from very different causes as the resulting gene repertoires of the two types of organisms differ tremendously. Indeed, the genome evolution of endosymbionts and obligatory pathogens is driven by two main processes which have mutually reinforcing effects on genome size and evolutionary rates. Being confined inside their host, these bacteria have tiny population sizes and are regularly bottlenecked at each host generation or at each new host infection. Consequently, they experience a strong genetic drift [28] involving an increase in substitution rate. This acceleration results in the accumulation at random of slightly deleterious mutations in protein-coding genes [8,29] as well as in rRNA genes [29,30]. This genetic drift enhances the downsizing of the genome through inactivation and then elimination of potentially beneficial but dispensable genes. Among these, there have been a number of DNA-repair genes, the disappearance of which could have further increased the mutation rate [6,31-33]. Furthermore, a number of genes may be subject to a relaxation of purifying selection which is therefore rendered less effective in maintaining gene function. This relaxation particularly affects genes which have become useless because they are redundant in their host genome, such as genes involved in the biosynthesis of amino acids, nucleotides, fatty acids and even ATP [4-6,8,9,32]. Selection pressure is also reduced for genes involved in environmental sensing and regulatory systems, such as two-component systems, because of the much buffered environment offered by the host [6].

In the free-living genus *Prochlorococcus*, the very large size of field populations [34] means that these populations are subject to much lower genetic drift and their genomes are subject to much stronger purifying selection than are those of endosymbionts and pathogens [35]. Consequently, the observed accelerated rate of evolution probably results merely from the increase in the mutation rate, which in turn is probably due to the loss of DNA-repair genes, even if one should note that, in *P. marinus *SS120 only two such genes are missing (Table 3). We observed a similar acceleration of amino-acid substitutions for all functional categories (Figure 4). This finding is more consistent with a global increase in the mutation rate than with relaxed selection, the latter being unlikely to occur to the same extent at all loci. We also assume that most amino-acid substitutions that have occurred in *Prochlorococcus *proteins are neutral; that is, they have not altered protein function. Indeed, populations of the HL clade which, like MED4, have the most derived protein sequences of all *Prochlorococcus *species, appear to be the most abundant photosynthetic organisms in the upper layer of the temperate and inter-tropical oceans [16]. Such an ecological success would hardly be possible for organisms handicapped by a large number of slightly deleterious mutations, especially given the fact that most genes are single copy, and so compensation of gene function is generally not possible. The effect of the maintenance of a high level of purifying selection on counteracting deleterious substitutions is particularly obvious in the rRNA genes. Contrary to the protein-coding genes, relative rate tests did not show any significant differences in the rates of evolution of the 16S rRNA genes in the four marine picocyanobacterial genomes, and thus there is no evidence that either SS120 or MED4 could have accumulated mutations destabilizing the secondary structure of their 16S rRNA molecule. One noteworthy consequence of the acceleration in the rates of evolution of protein-coding genes in *Prochlorococcus *is that phylogenetic reconstructions based on protein sequences are biased. Indeed, this leads to much longer branches for these two strains than for MIT9313. The resulting tree topology most often does not support that obtained with the 16S rRNA gene, for which the molecular clock hypothesis holds true according to our analyses. Thus, rRNA genes are likely to be among the few genes that will give reliable estimates of the phylogenetic distances between *Prochlorococcus *strains.

If it is neither the relaxation of purifying selection nor an increase in genetic drift that has been the main factor causing *Prochlorococcus *genome reduction, an alternative possibility is that the latter could be the result of a selective process favoring the adaptation of *Prochlorococcus *to its environment. The apparently better ecological success in oligotrophic areas of *Prochlorococcus *species compared to their close relative *Synechococcus *[16,34], strongly suggests that the reduction of *Prochlorococcus *genome size could provide a competitive advantage to the former. Indeed, extensive comparisons of the gene complements of these two organisms show very few examples - at least among genes for which function is known - of the occurrence of specific genes in MED4 which could explain its better adaptation (data not shown). One noteworthy exception is the presence in *Prochlorococcus*, but not *Synechococcus*, of flavodoxin and ferritin, two proteins that possibly give *Prochlorococcus *a better resistance to iron stress. Apart from that, *Synechococcus *appears more like a generalist, in particular with regard to nitrogen or phosphorus uptake and assimilation [22], and should *a priori *be more suited to sustain competition. Hence, we assume that the key to the success of *Prochlorococcus *resides less in the development of a specific complex or pathway to cope better with unfavorable conditions than in the simplification of its genome and cell organization, which can allow this organism to make substantial economies in energy and material for cell maintenance.

The mere reduction in genome size *per se *is a potential source of substantial economies for the cell, as it reduces the amount of nitrogen and phosphorus, two particularly limiting elements in the upper part of the ocean, which are necessary, for instance, in DNA synthesis. Another advantage is that it allows a concomitant reduction in cell volume. It has been previously suggested (see, for example [36]) that, for a phytoplanktonic organism, a small cell volume confers two selective advantages by reducing self-shading (the package effect) and by increasing the cell surface-to-volume ratio, which can improve nutrient uptake. The first advantage would improve the fitness of the LL strains, whereas the second would offer an advantage to the HL strains living in nutrient-depleted surface waters. Finally, cell division is less costly for a small than for a large cell. On the basis of these observations, we assume that the major driving force for genome reduction within the *Prochlorococcus *radiation has been the selection for a more economical lifestyle. The bias toward an A+T-rich genome in MED4 and SS120 is also consistent with this hypothesis, as it can be seen as a way to economize on nitrogen. Indeed, an AT base-pair contains seven atoms of nitrogen, one less than a GC base-pair.

**Figure 3:**
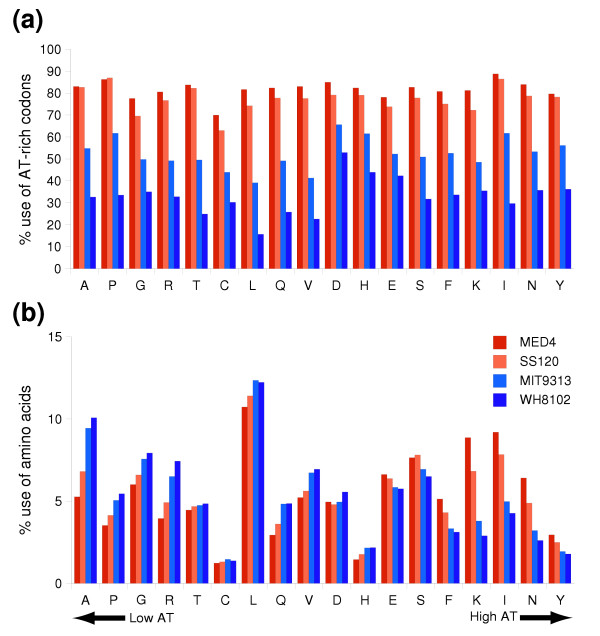
Influence of mutational bias in codon usage and amino-acid usage. **(a) **Percentage use of AT-rich codons in the four marine picocyanobacteria. Amino acids are ranked according to AT content of their respective codons. Methionine and tryptophan, which are both encoded by only one codon, have been discarded from the analysis. **(b) **Percentage use of amino acids in marine picocyanobacteria.

**Figure 4:**
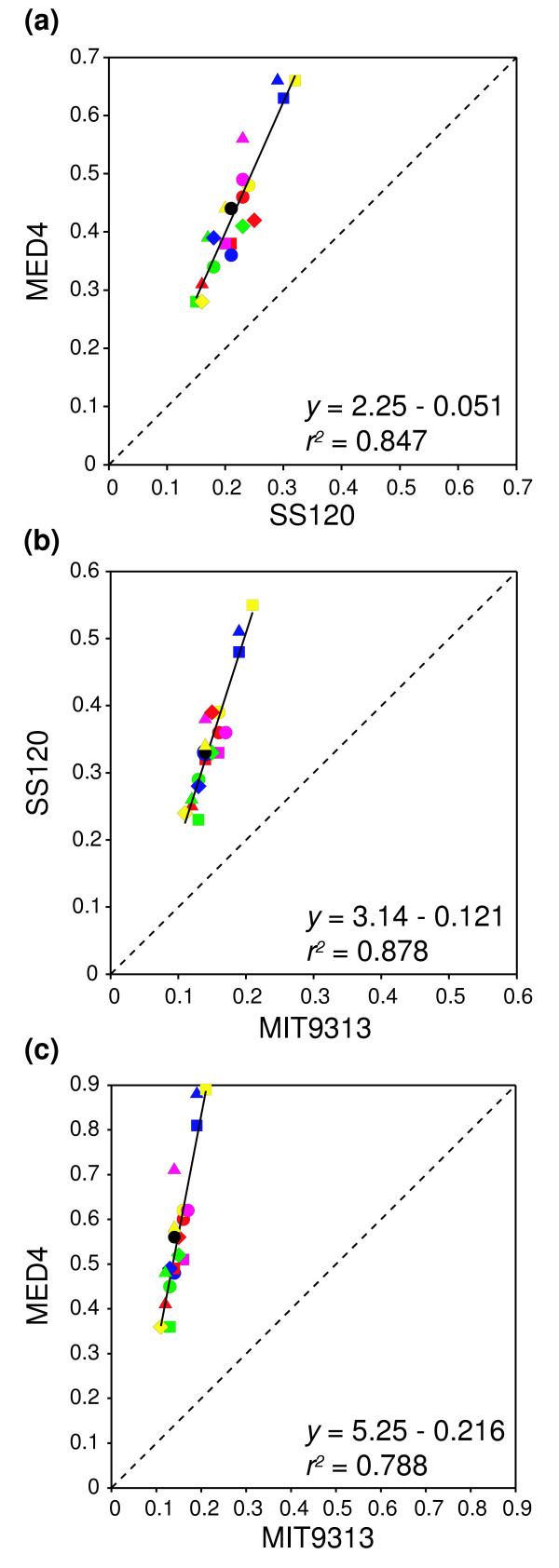
Amino-acid substitution rate per functional category. Branch lengths computed for each functional category between **(a) **MED4 and SS120, **(b) **SS120 and MIT9313 and **(c) **MED4 and MIT9313. In the three comparisons, branch-length values are aligned along a line with a slope much greater than 1, indicating that acceleration of the substitution rates occurs in every functional category. Axes represent the number of amino-acid substitutions per site. Red circle, amino-acid transport and metabolism; green circle, carbohydrate transport and metabolism; yellow circle, cell-cycle control; blue triangle, cell wall/membrane biogenesis; pink circle, coenzyme metabolism; red square, defense mechanisms; green square, energy production and conversion; yellow square, function unknown; blue square, general function prediction only; pink square, inorganic ion transport and metabolism; red triangle, intracellular trafficking; green triangle, lipid transport and metabolism; yellow triangle, nucleotide transport and metabolism; blue circle, posttranslational modification, protein turnover; pink triangle, replication, recombination and repair; red diamond, secondary metabolite biosynthesis, transport and catabolism; green diamond, signal transduction mechanisms; yellow diamond, transcription; blue diamond, translation; black circle, miscellaneous.

With this hypothesis in mind, we propose a possible scenario for the evolution of *Prochlorococcus *genomes. Using a rate of 16S rRNA divergence of 1% per 50 million years [37], one can estimate that the differentiation of these two genera is as recent as 150 million years, as the molecular clock hypothesis holds for this gene in *Prochlorococcus *and *Synechococcus*. The ancestral *Prochlorococcus *cells must have developed in the LL niche, a niche probably left free by other picocyanobacteria. Given the considerable difference in genome size between the LL strains MIT9313 and SS120, it appears that genome reduction itself must have started in one (or possibly several) lineage(s) within the LL niche some time after *Prochlorococcus *differentiation from its common ancestor with marine *Synechococcus *species. Why the selection has affected only one (or some?) and not all *Prochlorococcus *lineages remains unclear. Examination of the gene repertoire of *P. marinus *SS120 [13] suggests that this genome reduction must have concerned the random loss of dispensable genes from many different pathways. At some point during evolution, some genes involved in DNA repair have been affected; these would include the *ada *gene, which may be responsible for the shift in base composition, but also possibly several others, not necessarily involved in GC to AT mutation repair (see Table 3). Loss of these genes may have led to an increase in the mutation rate and therefore in the rate of evolution of protein-coding genes, accompanied by a more rapid genome shrinkage and a shift of base composition toward AT. It is worth noting that one likely consequence of this genome-wide compositional shift is the absence of the adaptive codon bias in the genomes of *Prochlorococcus *species MED4 and SS120. AT-rich codons are preferentially used whatever the amino acid (Figure 3a). Thus, codon usage in these genomes appears to reflect more the local base-composition bias than the selection for a more efficient translation through the use of optimal codons. The same conclusion has been drawn for other small genomes with high A+T content [28,38].

Later during evolution (around 80 million years ago, according to the degree of 16S rRNA sequence divergence between MED4 and SS120) one LL population which probably already had a significantly reduced cell and genome size must have progressively adapted to the HL niche and eventually recolonized the upper layer. How this change in ecological niche was possible is still hard to define. Comparison of the gene set that differs between the LL-adapted SS120 and the HL-adapted MED4 shows that very few genes might be sufficient to shift from one to the other niche, including a multiplication of *hli *genes [39] and the differential retention of genes which were present in the common ancestor of *Prochlorococcus *and *Synechococcus*, (such as the photolyases and cyanate transporters mentioned above) and were secondarily lost in the LL-adapted lineages.

## Conclusions

Genome evolution in the free-living genus *Prochlorococcus *has similar features to that in host-dependent prokaryotes: genome reduction, bias toward a low G+C content, acceleration in the evolution rate of protein-coding genes, and loss of DNA-repair genes. In contrast to the latter organisms, however, in *Prochlorococcus *this evolution does not appear to be the result of genetic drift or relaxed selection being exerted on some gene categories. Indeed, purifying selection is very efficient in *Prochlorococcus*, as rRNA genes have evolved at a similar rate in all genomes. Despite the decrease in G+C content and an accelerated rate of evolution of protein-coding genes, purifying selection must also act on these genes and avoid potentially deleterious mutations. We hypothesize that a reduction in genome size (which allows a concomitant reduction in cell size and substantial economies in energy and nutrients) can constitute a selective advantage for life in the open ocean, both at depths where photon energy is low and in surface waters where nutrients are scarce.

Genome shrinkage in *Prochlorococcus *has led to populations highly specialized to narrow ecological niches, at the expense of versatility and competitiveness in changing conditions. Indeed, not only is the distribution of the *Prochlorococcus *genus limited to low latitudes (40°N and 40°S, see [34]) but the different ecotypes are themselves more or less confined to a restricted part of the euphotic layer [40]; for example, they experience only limited changes in temperature and salinity. Paradoxically, because warm oligotrophic areas constitute a very large part of the world's oceans, the ecological niches (both LL and HL) occupied by *Prochlorococcus *species are huge, and thus this organism appears globally, despite its specialization, as one of the most successful oxyphototrophs on Earth.

## Materials and methods

### Genome sequence data

The complete genome sequences and annotations of *Prochlorococcus marinus *MED4, *P. marinus *SS120, *Prochlorococcus *sp. MIT9313 and *Synechococcus *sp. WH8102 (accession numbers: NC_005071, NC_005072, NC_005042 and NC_005070 respectively) were downloaded from the Genome division of the NCBI Entrez system. A few additional genes which were modeled in at least one genome and were present in the other genomes but not modeled (because of their small size, for example) were included in our dataset (see Additional data file 2).

### Alignment of whole genomes

Genome sequences translated in their six reading frames were aligned with the Promer program of the MUMmer 3.0 system [41].

### Codon and amino-acid usage

Codon usage was computed for every open reading frame (ORF) of each genome with the EMBOSS program cusp. Amino-acid usage was derived from the results produced by cusp.

### Identification of orthologous proteins

We used a sequence-similarity based approach which is similar to the procedure used for the cluster of orthologous groups (COGs [42]). For each genome pair, all-against-all BLAST [43] comparisons were performed using protein sequences and reciprocal genome-specific best hits were identified. We considered genes as being probable orthologs when they were included in groups of size four in which each gene was the best hit of the three others. From similarity searches against the COG database, orthologs were assigned to functional categories according to those defined for the COG system. Because of the lack of a particular category for photosynthesis genes, the latter were assigned to the 'energy production and conversion' COG category. Other genes which fell into more than one of the 19 COG categories have been assigned to a supplementary category called 'miscellaneous'.

### Phylogenetic branch length estimations

Protein sequences from each of the groups of four orthologous genes were aligned using ClustalW [44] with default parameters. After exclusion of all gap sites, individual alignments were concatenated in one super-alignment of 388,120 sites. Gamma distances [45] with an alpha parameter of 1 were estimated between each pair of sequences of the super-alignment. Phylogenetic branch lengths were calculated from distances with the ordinary least-squares method [45]. Relative rate tests (two-cluster test and Branch length test) were applied in order to test the constancy of amino-acid substitution rates between the three *Prochlorococcus *genomes (hypothesis of the molecular clock). The same analysis was applied to orthologs of each functional category.

### Estimate of synonymous and nonsynonymous substitution rates

Nucleotide sequences of each group of orthologs were aligned with Protal2dna according to alignments of their corresponding amino-acid sequences [46]. Pairwise estimates of the synonymous (*d*_S_) and non-synonymous (*d*_N_) substitution rates were obtained from the Yn00 program of the PAML 3.13 package [47].

## Additional data files

The following additional data are available with the online version of this article. Additional data file 1 lists the orthologous genes classified by functional category. Orthologous genes were assigned to the functional categories of COG system. Photosynthesis genes were assigned to the 'energy production and conversion' COG category. Genes falling in more than one of the 19 COG categories have been assigned to a supplementary category called 'miscellaneous'. Additional data file 2 is a fasta file of orthologous genes which were modeled in at least one genome and present but not modeled in the other genomes.

## Supplementary Material

Additional data file 1 The orthologous genes classified by functional category Click here for additional data file

Additional data file 2 A a fasta file of orthologous genes which were modeled in at least one genome and present but not modeled in the other genomes Click here for additional data file

## Figures and Tables

**Table 1:** General features of the genomes of the four marine picocyanobacteria used in this study

Genome	Size (Mbp)	GC%	Number of protein-coding genes
*P. marinus *MED4	1.66	30.8	1,716
*P. marinus *SS120	1.75	36.4	1,882
*Prochlorococcus *sp. MIT9313	2.41	50.7	2,273
*Synechococcus *sp. WH8102	2.43	59.4	2,525

**Table 2:** Number and percentage of orthologous genes per functional category

Category	Number of genes	% of orthologs
Amino-acid transport and metabolism	94	7.2
Carbohydrate transport and metabolism	50	3.8
Cell-cycle control	17	1.3
Cell wall/membrane biogenesis	55	4.2
Coenzyme metabolism	99	7.6
Defense mechanisms	14	1.1
Energy production and conversion	106	8.1
Function unknown	269	20.6
General function prediction only	116	8.9
Inorganic ion transport and metabolism	47	3.6
Intracellular trafficking	13	1.0
Lipid transport and metabolism	25	1.9
Nucleotide transport and metabolism	39	3.0
Posttranslational modification, protein turnover	59	4.5
Replication, recombination and repair	51	3.9
Secondary metabolite biosynthesis, transport and catabolism	6	0.5
Signal transduction mechanisms	11	0.8
Transcription	26	2.0
Translation	127	9.7
Miscellaneous	82	6.3

**Table 3:** DNA-repair genes missing only in *P. marinus *MED4 or in both MED4 and SS120

Gene	COG	Product	MED4	SS120	MIT9313	WH8102
***ada/ogt***	0350	6-O-methylguanine-DNA methyltransferase	-	-	PMT0269	SYNW1680
***mutY***	1194	A/G-specific DNA glycosylase	-	Pro1789	PMT0135	SYNW0115
*recQ*	0514	Superfamily II DNA helicase	-	-	PMT0189	SYNW1958
*recJ*	0608	Single-stranded DNA-specific exonuclease	-	Pro0984	PMT0761	SYNW1206
*exoI/xseA*	1570	Exonuclease VII large subunit	-	Pro0111	PMT1641	SYNW2181
*xseB*	1722	Exonuclease VII small subunit	-	Pro0112	PMT1642	SYNW2182
-	0494	NUDIX hydrolase family	-	-	PMT1026	SYNW1334

Genes in bold are involved in repair of G:C to A:T mutations.
